# Spectral Preprocessing Combined with Deep Transfer Learning to Evaluate Chlorophyll Content in Cotton Leaves

**DOI:** 10.34133/2022/9813841

**Published:** 2022-08-16

**Authors:** Qinlin Xiao, Wentan Tang, Chu Zhang, Lei Zhou, Lei Feng, Jianxun Shen, Tianying Yan, Pan Gao, Yong He, Na Wu

**Affiliations:** ^1^College of Biosystems Engineering and Food Science, Zhejiang University, Hangzhou 310058, China; ^2^Key Laboratory of Spectroscopy Sensing, Ministry of Agriculture and Rural Affairs, Hangzhou 310058, China; ^3^School of Information Engineering, Huzhou University, Huzhou 313000, China; ^4^College of Mechanical and Electronic Engineering, Nanjing Forestry University, Nanjing 210037, China; ^5^Hangzhou Raw Seed Growing Farm, Hangzhou 311115, China; ^6^College of Information Science and Technology, Shihezi University, Shihezi 832000, China

## Abstract

Rapid determination of chlorophyll content is significant for evaluating cotton's nutritional and physiological status. Hyperspectral technology equipped with multivariate analysis methods has been widely used for chlorophyll content detection. However, the model developed on one batch or variety cannot produce the same effect for another due to variations, such as samples and measurement conditions. Considering that it is costly to establish models for each batch or variety, the feasibility of using spectral preprocessing combined with deep transfer learning for model transfer was explored. Seven different spectral preprocessing methods were discussed, and a self-designed convolutional neural network (CNN) was developed to build models and conduct transfer tasks by fine-tuning. The approach combined first-derivative (FD) and standard normal variate transformation (SNV) was chosen as the best pretreatment. For the dataset of the target domain, fine-tuned CNN based on spectra processed by FD + SNV outperformed conventional partial least squares (PLS) and squares-support vector machine regression (SVR). Although the performance of fine-tuned CNN with a smaller dataset was slightly lower, it was still better than conventional models and achieved satisfactory results. Ensemble preprocessing combined with deep transfer learning could be an effective approach to estimate the chlorophyll content between different cotton varieties, offering a new possibility for evaluating the nutritional status of cotton in the field.

## 1. Introduction

Cotton is one of the most important economic crops due to its excellent natural properties. The growth and development of cotton are inseparable from photosynthesis. Chlorophyll (Chl) is the most important organic molecule in the photosynthesis of green plants and a vital component of leaf chloroplasts [1]. Chl content can be used to assess the process of photosynthesis and the potential maximum CO_2_ assimilation rate [2], and determining Chl content is an important part of the evaluation of cotton's physiological status. The changes in Chl content reflect the plant's photosynthetic capacity and indirectly reveal their nutritional status, senescence, and disease stress [3]. Hence, fast and accurate detection of Chl content is essential. The conventional methods for Chl content detection mainly include ultraviolet-visible spectrophotometry [4] and high-performance liquid chromatography [5]. Although these methods are feasible to measure Chl content with good reproducibility and high accuracy, defects such as laborious, poor timeliness, and irreversible sample damage limited their application. In recent years, nondestructive methods have been developed to detect internal components of plants. Hyperspectral technology has been widely studied and has proven effective in determining Chl content in various plants [4, 6–8].

There are two main approaches for the research on the detection of leaf Chl based on hyperspectral technology: building models based on direct spectral data or vegetation index. For the former, the model is established based on the full spectra or a few bands with characteristic spectral responses [7, 9–11]. For the latter, the model is constructed based on multispectral vegetation indices established according to the characteristic bands [12, 13]. No matter which method is used, establishing a multivariate model is a commonly used approach for Chl content detection based on hyperspectral technology. However, hyperspectral technology coupled with multivariate analysis has some problems in practical applications. The acquired spectra are affected by various factors, such as the noise in the measurement environment, the difference in chemical and physical properties of the samples, and even the different instruments [14]. Variations in feature spaces and data distributions may make the model built based on the previous batch of samples hard to be used for the next. It is also difficult to apply the model established by the same plant species between different varieties or measurement conditions [15]. A typical way to solve this problem is to develop a new model when the samples or measurement conditions are changed. However, this approach is not a priority since it requires collecting many new samples and is costly and time-consuming. Making corrections in which the variations are fully considered can help the model be reused in the new dataset and reduce the cost of constructing new models. Some calibration transfer methods have been proposed to solve the problem that the model based on the data obtained from a specific instrument fails to be reused for another, such as segmented direct standardization (PDS) [16], direct standardization (DS) [17], and some other methods [18]. Then, calibration transfer is developed to evolve model adaptation across different datasets [19]. There are two approaches to achieve calibration transfer. The first one is to reduce the differences between data in different domains, such as spectral preprocessing. And make the model learn general representations that cover the main data features. Spectral preprocessing is a commonly used calibration transfer method as the first step of spectral processing analysis [20]. Spectral preprocessing can reduce and eliminate the influence of various nontarget factors, enhance spectra commonality, and simplify subsequent analysis and modeling calculation processes to improve models' predictive ability and robustness. The performance of the models based on spectra preprocessed by different pretreatment varies. In general, optimal spectral pretreatment selection is empirical and tentative. Each pretreatment is suitable for certain situations, and detailed information can be found in the literature [20]. Another one is to use additional algorithms to calibrate data between different domains. However, this type of calibration transfer algorithm requires standard samples. Generally, more standard samples can achieve better performance. However, it is often hard to collect spectra of standard samples under varied conditions. Besides, in regression tasks, the data distributions of standard samples are also of great influence in performing a good calibration transfer. It is also a big challenge to select the standard sample with appropriate distribution of statistical values. Therefore, realizing simple and effective calibration transfer between different datasets remains an urgent problem to be overcome.

Transfer learning has been recently used to transfer knowledge between different domains. At present, transfer learning has been successfully applied in recognition tasks in computer vision [21, 22] and the classification of hyperspectral images [23, 24]. As for the application of transfer learning in spectra analysis, Feng et al. [15] used transfer learning methods to achieve disease classification for different rice varieties. The fine-tuning method yielded the highest accuracy in the majority of transfer tasks. Liu et al. [25] employed a pretrained CNN based on spectra measured in laboratory conditions and explored the potential of using transfer learning to make the model adaptable to airborne spectra. Puneet et al. [26] developed a pretrained CNN and transferred the model between different measurement instruments using the fine-tuning method. Recently, Zhang et al. [27] applied fined-tune transfer learning and amplitude- and shape-enhanced 2D correlation spectrum and achieved the knowledge transfer between simulated dataset and field observation, improving the inversion accuracy of winter wheat Chl content under different field scenarios. These studies show the great potential of deep transfer learning in calibration transfer.

Although great progress has been made in detecting chlorophyll content in plant leaves, there is still a lack of research on the adaptability of models under different conditions. Therefore, the main purpose of this research is to investigate the feasibility of spectral preprocessing combinations and deep transfer learning for the calibration transfer of Chl content prediction models in cotton leaves. The specific objectives include the following: (1) compare the leave spectral characteristics of the whole growth cycle of two cotton cultivars; (2) explore the optimal spectral preprocessing method for model calibration transfer between cultivars; (3) establish a CNN model for Chl content prediction based on the optimal preprocessed spectra and apply the CNN model trained on a specific variety of cotton to another with fine-tuning; and (4) use the saliency map to visualize the key wavelengths captured by the fine-tuned CNN.

## 2. Materials and Methods

### 2.1. Sample Preparation

An experiment was carried out from April to October in 2021 at the Hangzhou Raw Seed Growing Farm (30°22′58.85″ N, 119°56′7.80″ E), Hangzhou, Zhejiang province, China. Six nitrogen rates (0, 120, 240, 360, 480, and 278 kg/hm^2^) were set in this experiment. Two cotton cultivars were tested: Lumianyan 24 (LMY24) and Xinluzao 53 (XLZ53). Cotton seeds were provided by Shihezi University, Shihezi, the Xinjiang Uygur autonomous region, China. All the treatments were arranged in the randomized complete block design with 3 replicates. A total of 36 plots were sown, and individual plots were sized 4 × 2 m. Three cotton rows consisted of a spacing distance of 0.6 m. The width of the irrigation ditch between the two adjacent plots is 1 m. In addition to nitrogen, the dosage of phosphate fertilizer (P_2_O_5_) and potassium fertilizer (K_2_O) was 150 kg/hm^2^.

### 2.2. Spectra Acquisition

The experiment was conducted at five growth stages: bud stage (stage 1), flowering stage (stage 2), boll-forming stage (stage 3), peak boll-forming stage (stage 4), and initial flocculating stage (stage 5). Three plants in each plot were randomly selected. The leaves at the different leaf positions were sampled. Leaf spectra were acquired in reflectance mode with a spectroradiometer (Fieldspec4, Analytical Spectral Devices (ASD), Boulder, CO USA). The spectral resolution was 3 nm for the visible and near-infrared region (350~1000 nm) and 8 nm for the shortwave-infrared region (1000~2500 nm). Measurement was carried out using a leaf clip, which provides a calibrated light source. Before leaf spectra collection, reflectance calibration was performed with standard white reference. Leaf midrib and edges were avoided when measuring. Each measurement consisted of 5 scans, and the average value was recorded as the measurement value. The spectra of three different regions of each leaf were recorded, and their mean was taken as the leaf spectrum. Removing the head of spectra with high noise levels, the spectra within the range of 430-2500 nm were kept and used for subsequent analysis. It is worth mentioning that spectra acquired at the bud stage, flowering stage, and boll-forming stage were captured in the field. The spectra acquired at the peak boll-forming stage, and initial flocculating stage were captured in the laboratory environment.

### 2.3. Measurements of Chl Content

**Figure 1:**
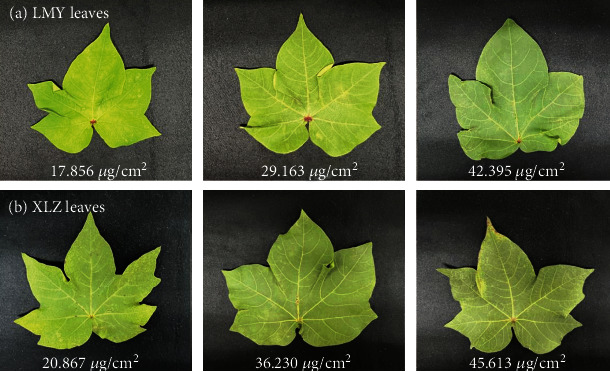
Cotton leaves with different Chl content.

After spectra acquisition, each leaf was placed in a labeled and sealed bag stored in an icebox with a temperature of about 2 °C temporarily. The leaves were quickly transported to the laboratory (Zhejiang University, Zhejiang Province, China) and were tested for Chl content. Leaf discs were collected with a hole punch with a diameter of 0.86 cm. Three leaf discs of each leaf were collected and immersed in 4 mL 95% ethanol. The leaf discs tubes were placed in a dark environment for about 48 h until the leaves turned white and the Chl was completely leached. A spectrophotometer (Epoch, BioTek Instruments, Winooski, United States) was used to measure the absorbance of the extracted solution at the wavelengths of 470, 649, and 665 nm, which could be utilized to calculate the Chl content according to the formula in the literature [28]. The cotton leaves with different Chl content were shown in Figure 1.

### 2.4. Data Analysis Methods

#### 2.4.1. Outliers Detection

In the whole experiment, the number of leaves with valid Chl content values for the variety LMY 24 and XLZ53 were 789 and 795, respectively. To conduct better modeling analysis, the method of combining principal component analysis and Hotelling T^2^ mentioned in literature [29] and BoxPlot were used to remove outliers before data processing. As a result, twenty outliers were removed for LMY24 and 26 for XLZ53. Therefore, the number of samples for LMY24 and XLZ53 used for further analysis were both 769.

#### 2.4.2. Spectral Preprocessing

Some common spectral preprocessing methods and their combinations have been used to reduce and eliminate unwanted variation and improve the predictive ability and robustness of the model. The methods applied in this study include standard normal variate transformation (SNV), detrending, multiplicative scatter correction (MSC), and first-derivative (FD). SNV is used to eliminate the effect of particle size, surface scattering, and optical path changes on the spectra [30]. Detrending is used in conjunction with SNV to correct the baseline drift of diffuse reflectance spectrum. MSC has been proved linearly related to SNV [31], and its role is similar to that of SNV [30]. In addition, the derivation is commonly used to improve spectral resolution by calculating the adjacent slope wavelengths. In general, smoothing is usually used before derivation to reduce its influence on the signal-to-noise ratio. In this paper, Savitzky-Golay smoothing was used before FD preprocess. More detailed information on SNV, detrending, MSC, and derivation can be found in [31, 32]. In addition to using some preprocessing algorithms individually, some combinations in which the subsequent transformation supplemented the previous method were also considered.

#### 2.4.3. Convolutional Neural Network and Transfer Learning Method

**Figure 2:**
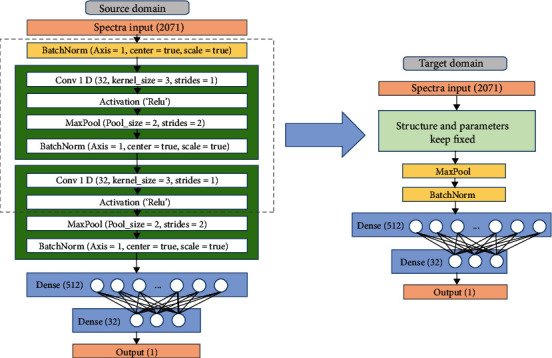
The architectures of the CNN model and the flowchart of fine-tuning transfer.

As one of the representative deep learning algorithms, convolutional neural network (CNN) achieves feature and representation learning through convolution operation. It shows excellent performance in various spectral classification and regression tasks [15, 33–35]. In this study, a one-dimensional CNN architecture was constructed for the regression task, and its structure is shown in Figure 2. Firstly, a batch normalization layer was added as a standardization process for forcing the distribution of input values of the convolution layer back to the standard normal distribution with a mean of 0 and variance of 1. Then, two convolution blocks were included, in which a convolution layer and max-pooling layer were set, followed by the batch normalization layers. Convolutional kernels of different sizes help extract deep spectral features, and stacked convolutional layers enhance the ability to extract features at abstraction levels [36]. The number of filters, kernel size, and strides of the two convolution layers were set as 32, 3, and 1, respectively. The rectified linear unit (ReLU) served as the activation for calculating the outputs of the convolutional layers. By utilizing the max-pooling layers, downsampling and dimension reduction were performed to form the features for the next layers. Then, two fully connected layers were applied. Each of them was composed of 512 and 32 neurons, respectively. At the end of the network, another fully connected layer was used for output.

The L2 loss function and an adaptive moment estimation (Adam) optimizer were employed to train the CNN regression model. A scheduled learning rate was used in the training phase. In the beginning, the learning rate was set as 0.005. The learning rate was reduced ten times after every 200 epochs. According to this rule, the training process was terminated once the loss stabilized. The batch size was set to 64.

In transfer learning, a source domain *𝒟*_*S*_, a target domain *𝒟*_*T*_, and a task *𝒯* = {*y*, *f*(.)} were defined. The source domain *𝒟*_*S*_ and the target domain *𝒟*_*T*_ are pairs of {*x*_*i*_, *p*_*i*_}, where *x*_*i*_ is the feature space and *p*_*i*_ is the probability distribution corresponding to *x*_*i*_. Generally, the feature space or the probability distribution of the source domain *𝒟*_*S*_ and the target domain *𝒟*_*T*_ varies. The *y* of task *𝒯* indicates a label space, and the *f*(.) implies a predictive function. When *𝒯* was conducted, *f*(.) constructed a model using {*x*_*i*_, *p*_*i*_} in the domain. The goal of transfer learning is to improve the performance of the predictive function in the target domain *𝒟*_*T*_ with using the knowledge learned from the source domain *𝒟*_*S*_ [37]. Fine-tuning is a common method in deep transfer learning. In this study, fine-tuning method was used to build a model for the Chl content detection of cotton leaves that could be transferred between different cultivars. The fine-tuning method takes the target dataset as the new input of the pretrained model and fine-tunes the weight of original networks. As shown in Figure 2, the spectra of the source domain were used to train a CNN model, and the parameters of the layers in the dotted box were kept frozen. Then, the spectra of the target domain were used to fine-tune the pretrained model.

#### 2.4.4. Conventional Regression Models

Partial least squares (PLS) and squares-support vector machine regression (SVR) models were built using the average spectra of each leaf and its corresponding Chl content. PLS is widely used in regression modeling for high-dimensional datasets. PLS can fit the linear regression relationship between spectral variables and Chl content values. Unlike normal multiple linear regression, PLS takes advantage of the useful information in each band and avoids severe collinearity between variables [33]. SVR is a popular machine learning algorithm with a good generalization ability and helps to solve the high dimensionality problem. SVR maps variables and target values to a high-dimensional space through nonlinear transformation and constructs a linear decision function to achieve linear regression [38]. The kernel function is especially essential for model construction. Radial basis function (RBF) shows powerful processing capabilities for nonlinear problems [39]. RBF kernel was used in this study, and the combination of the regularization parameter *c*, and the kernel function parameter *g* was optimized by grid search. The searching range of *c* and *g* were assigned from 10^−7^~10^7^ and 10^−9^~10^1^, respectively. In this study, five-fold cross-validation was adopted for PLS and SVR models.

#### 2.4.5. Visualization

Visualization transforms data into images for the intuitive presentation that contributes to a clearer understanding. The saliency map is a popular technique for model visualization, and it can reflect the contribution of each variable to model performance. It is widely used in two-dimensional image classification due to its advantages of intuitively showing the importance of each pixel in images. Recently, it has been extended to analyzing multidimensional data [15, 40]. In this study, we made a simple modification based on the method proposed in Feng's study [15] and made it suitable for regression problems. Firstly, we trained the CNN model and obtained the predicted value of Chl. Then, we calculated the error rate of prediction corresponding to the following equation:

(1) $$\begin{array}{c} \text{the }error\;rate=\left|\frac{\text{the }predicted\;value-\text{the }measured\;value}{\text{the }measured\;value}\right|*100\%. \end{array}$$

The samples with an error rate within 5% were taken as “correctly predicted samples.” The saliency map was computed based on the “correctly predicted samples.” The computed gradient reflects the influence of each band on the correct classification. The higher the gradient value, the more influence it has on the correct prediction. Next, the wavelengths for each “correctly predicted sample” were sorted in descending order of the absolute value of the corresponding gradient. The first 100 critical wavelengths of each “correctly predicted sample” were selected, and the frequency of each wavelength was counted. Finally, the saliency map was plotted based on the frequency of the important bands.

#### 2.4.6. Software and Model Evaluation

Outlier detection was conducted in MATLAB R2015b (The MathWorks, Natick, MA, USA). SNV, MSC, and detrending were performed in the Unscrambler X 10.1 (Camo AS, Oslo, Norway). FD was undertaken in MATLAB R2015b (The MathWorks, Natick, MA, USA). For the model establishment, the construction of the PLS model was performed in the Unscrambler X 10.1 (Camo AS, Oslo, Norway). SVR was carried out in the scikit-learn 0.23.1 (Anaconda, Austin, TX, USA) using python 3.1. The CNN model and fine-tuning were conducted in MXNet1.4.0 (MXNetAmazon, Seattle, WA, USA).

The coefficients of determination (*R*^2^) and root mean square error (RMSE) of calibration, validation and prediction set were calculated to evaluate model performance. The *R*^2^ of a robust model should approach 1, while the RMSE is close to 0.

## 3. Results

### 3.1. Spectra Profiles

**Figure 3:**
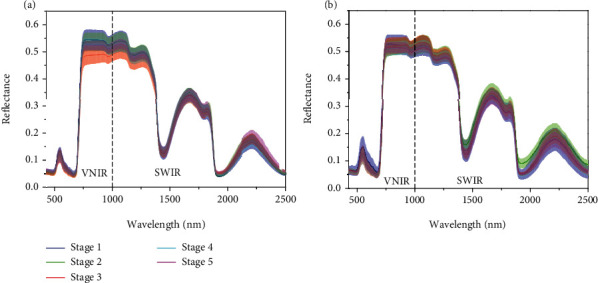
The average spectra with standard deviation (SD) of leaves of two cotton varieties (a) LMY 24 and (b) XLZ 53 captured at five growing stages.

The average spectra with standard deviation of leaves of two cotton cultivars (LMY 24 and XLZ53) captured at five growing stages are presented in Figure 3. It can be observed that the change tendencies of the cotton leaves of both cultivars were the same. Four peaks (550, 1650, 1820, and 2225 nm) and three valleys (670, 1432, and 1950 nm) were observed in spectral curves. The reflectance peak at 550 nm and the valley around 670 nm were caused by the Chl absorption [41]. The peak near 1650 nm was designated as the first overtone of the C–H stretch, and the peak around 1820 nm was assigned to the combination of O-H and 2 C-O stretches [42]. The bands near 1432 nm had been attributed to the second overtone of the N-H stretch [42]. Moreover, the wavelengths around 1950 nm and 2225 nm were assigned to the second overtone of the C-O stretch [42] and the combination of the asymmetrical N-H stretch and NH_2_ rocking [43], respectively. In addition, some distinct differences between samples from different stages were shown in the range of 520~580, 750~1350, 1500~1850, and 2200~2400 nm. Such localized differences can arise from a range of factors such as differences in intrinsic components, measurement environment, and operators. The main point is that compositional differences unrelated to interference are the basis for establishing the detection model of Chl content.

### 3.2. Regression Models for All Cotton Leaves

PLS and SVR models were established based on all leaves. The samples of LMY24 and XLZ53 were pooled and then sorted according to the ascending order of the Chl content. The first and third samples of every three were selected into the calibration set, and the remaining ones were divided into the prediction set. The detailed information on the calibration set and the prediction set are shown in Table 1. The regression results based on all the leaves are shown in Table 2. It can be seen that PLS and SVR models gained an R^2^_P_ over 0.76. The SVR model outperformed the PLS model, with the R^2^_P_ and RMSEP of 0.822 and 3.472. These results indicated that it is feasible to establish a model for Chl content prediction of cotton leaves based on visible and near-infrared spectra. The nonlinear model performed better, which may attribute to more nonlinear patterns in the correspondence between the spectrum and chlorophyll content. In the above analysis, both varieties of cotton leaves were involved in the modeling, and the samples used for prediction were also from these two varieties. However, it is always necessary to transfer the established model to new cultivars in practice. Therefore, the adaptability and transfer performance of the model should be fully discussed.

### 3.3. Effects of Different Pretreatments on Model Transfer

The transfer performance of the models among different cultivars and the influence of spectral preprocessing were explored. The samples of one cotton cultivar were as calibration set, and the samples of the other were as prediction set. PLS and SVR models based on one cotton cultivar were used to predict another. The results are shown in Tables 3 and 4.

It can be seen that when none preprocessing method was applied, compared with R^2^_CV_ and RMSECV, the R^2^_P_ and RMSEP of all PLS and SVR models decreased and increased with inconsistent magnitude, respectively. Specifically, taking the SVR model as an example, when LMY24 was the source domain, the R^2^_CV_ and RMSECV of LMY24 were 0.867 and 2.981, respectively, while the R^2^_P_ and RMSEP of XLZ53 were 0.700 and 4.572. When XLZ53 was transferred to LMY24, the R^2^_CV_ and RMSECV of XLZ53 were 0.896 and 2.687, respectively, while the R^2^_P_ and RMSEP of XLZ53 were 0.618 and 5.047. This phenomenon indicated that when the model built based on LMY24 was transferred to XLZ53, the prediction performance was better than that established on XLZ53 aiming to predict LMY24. It indicates that the containment relationship of spectral signals varies from different varieties of cotton leaves. It can be inferred that the spectral characteristics of LMY24 have a higher containment degree than those of XLZ53. Therefore, exploring suitable methods to make the model based on a single cultivar applicable to other cultivars is necessary.

Table 3 shows the prediction results of PLS and SVR models built with spectra of LMY24 for XLZ53 prediction. The performance of the models established by different preprocessed spectra varies. In all PLS models for Chl content prediction of XLZ53, the model based on transformed spectra by FD + SNV outperformed other models, with R^2^_P_ increasing by 14.2% and RMSEP declining by 26.2% based on the raw spectra modeling. Regarding SVR models, the results based on FD + MSC preprocessing were slightly better than those based on FD + SNV pretreated spectra. The prediction results of PLS and SVR models built with spectra of XLZ53 for Chl content prediction of LMY24 are presented in Table 4. The PLS and SVR models based on the spectra pretreated by FD + SNV yielded the best results. Compared with the model built on raw spectra, the R^2^_P_ of the PLS model and SVR model based on FD + SNV pretreated spectra increased by 17.8% and 4.7% and RMSEP decreased by 14.8% and 3.8%, respectively. Based on the above analysis, FD + SNV demonstrated great generalization ability and was selected as the optimal preprocessing method.

### 3.4. Regression Models Using Spectral Preprocessing and Transfer Learning

In order to establish a pretraining CNN model based on the spectra of a single cultivar, the leaves of each cotton cultivar were redivided into the calibration set, validation set, and prediction set in a ratio of 3 : 1 : 1. Firstly, a pretrained CNN model based on one cotton cultivar was established, and then the pretrained CNN was fine-tuned using the calibration set of another cultivar. Before modeling, FD + SNV pretreatment was applied for the spectra of both cultivars. The results are shown in Table 5. All the models built with preprocessed spectra were superior to the corresponding models established with the spectra without pretreatment. In addition, the performance of the fine-tuned CNN was better than that of PLS and SVR models regardless of preprocessing. The phenomenon is consistent with the above results in 3.3, indicating the effectiveness of pretreatment, as well as the effectiveness of fine-tuning.

When LMY24 was the source domain, the fine-tuned CNN established by preprocessed spectra outperformed the PLS and SVR model. Its R^2^ were 0.909, 0.850, 0.870, and RMSE were only 2.505, 3.248, and 3.020 for calibration set, validation set, and prediction set of the target dataset. Compared with the PLS model, the RMSE was reduced by 30.42%, 25.49%, and 26.31%. A similar large drop was also observed in comparison with the SVR model. When XLZ53 was the source domain, the performance of the fine-tuned CNN based on FD + SNV pretreatment performed best. The R^2^ were up to 0.889, 0.835, and 0.822, and the RMSE were 2.708, 3.332, and 3.460 for calibration set, validation set, and prediction set of the target domain, respectively. Whether the source domain was LMY24 or XLZ53, the fine-tuned CNN combined with FD + SNV pretreatment yielded the best results. It demonstrated the superior performance of combining transfer learning and spectral signal preprocessing for Chl content prediction. Besides, to further explore the effectiveness of fine-tuning, CNN models were also fine-tuned with a smaller dataset of the target domain. The smaller datasets only contain half of the samples in the original calibration set, and the validation set, and prediction set remain unchanged. As shown in Table 5, the result of the fine-tuned CNN using a smaller set was similar to or slightly lower than that using a full calibration set, regardless of the preprocessing. Fine-tuned CNN with a small dataset was still superior to PLS and SVR models in both transfer tasks. The results show that fine-tuning is conducive to the knowledge transfer of different datasets. Satisfactory results can be obtained even if the dataset of the target domain used for training is relatively small.

### 3.5. Saliency Map

**Figure 4:**
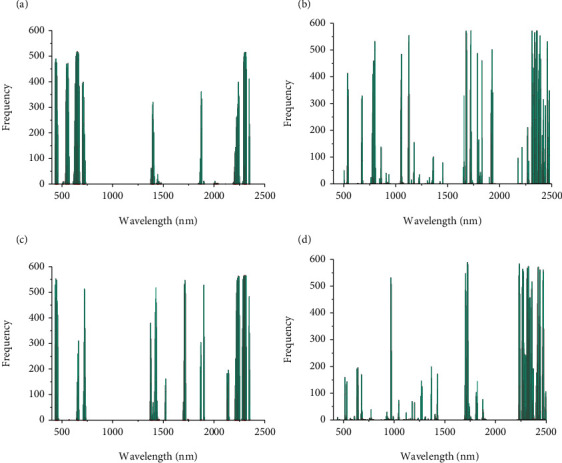
Saliency map after transfer learning regarding two cotton varieties. (a)–(d) Key wavelengths of for the Chl content determination by different CNN: (a) the fine-tuned CNN using raw spectra (LMY24→XLZ53); (b) the fine-tuned CNN using FD + SNV preprocessed spectra (LMY24→XLZ53); (c) the fine-tuned CNN using raw spectra (XLZ53→LMY24); and (d) the fine-tuned CNN using FD + SNV preprocessed spectra (XLZ53→LMY24).

The saliency map was used for visualizing the frequency of the critical wavelengths for the Chl content determination by fined-tuned CNN using different processed spectra. As shown in Figures 4(a) and 4(c), the critical bands identified by the fine-tuned CNN using raw spectra are almost located in the same range. When LMY24 was the source domain, and XLZ53 was the target domain, the important wavelengths captured by the fine-tuned CNN using raw spectra were mainly concentrated in the range of 432~463 nm, 532~571 nm, 607~674 nm,702~731 nm, 1374~1411 nm, 1859~1879 nm, 2198~2251 nm, and 2287~2319 nm. These spectral ranges greatly overlapped the located range by the fined-tuned CNN using raw spectra in the transfer task from XLZ53 to LMY24. These ranges include bands that have been identified to be closely related to Chl, such as the bands in the red edge (700~750 nm), red (630~690 nm), and green band (500~580 nm) regions [44, 45]. Some of the identified key wavelengths of Chl by fine-tuned CNN (550 nm and 717 nm) were also found to be associated with Chl detection by other studies [46] [47]. Moreover, the important wavelengths located in the near-infrared range (1380 nm [48], 2225 nm [43], 1325~1575 nm and 2125~2275 nm [49]) were considered to be sensitive to nitrogen. In addition, the frequency of the wavelengths identified by fine-tuned CNN using FD + SNV processed spectra are shown in Figure 4 (b) and (d). A similar intersection of the effective wavelengths was observed in the transfer tasks between two varieties. Different from the bands located by the fine-tuned CNN based on raw spectra, quite a lot of essential wavelengths found by the fine-tuned CNN based on preprocessed spectra were in the near-infrared region between 2264 and 2479 nm, where various nitrogen-containing bonds were likely to be responsible for the spectra variation [43]. This phenomenon presented in this study is consistent with the results described by Yoder [50]. Compared with raw spectra, higher correlations between wavelengths in the near-infrared region and Chl were observed with the first-difference transformation (approximating first derivatives) [50]. Overall, the similarity of high-frequency wavelengths located by fine-tuned CNN between two varieties indicated that fine-tuning could realize the transfer learning of main features between data in similar domains.

### 3.6. Comparison between the Effect of Different CNN Architectures on Fine-Tuning

The above results demonstrated that spectral preprocessing combined with deep transfer learning could achieve effective model transfer between different domains. However, the impact of different CNN architectures on the performance of fine-tuned models deserves further exploration. We evaluated six different CNN architectures: four self-developed CNNs, modified AlexNet, and VGGNet. The convolutional layers of AlexNet and VGGNet were modified to be suitable for one-dimensional input, and the number of hidden layers of VGGNet was decreased from 16 to 9. The different CNN architectures are shown in Table 6. CNN1 is the model with the simplest structure. The model complexity gradually increases from CNN1 to VGGNet-9, and VGGNet-9 is the model with the most complex architecture. In the transfer learning process, all layers of the pretrained CNN are frozen, except for the last two fully connected layers. The whole training process of the model remains the same as the method introduced in Section 2.4.3. Ten training processes were conducted for each architecture. The results of the three smallest RMSE values of the prediction set of the target domain were averaged as the indicator.

Table 7 shows the results of fine-tuned models using different CNN architectures. It can be observed that the CNN1 architecture performed significantly well, while the VGGNet-9 architecture had a less satisfactory performance. When the source domain was XLZ53, the RMSE of the prediction set tended to increase with the increased complexity level of CNN architecture. When the source domain was LMY24, the fine-tuned CNN1 yielded the best results than other CNN architectures with more complex levels. This phenomenon exhibited that complex CNN architectures are unsuitable for the Chl detection model transfer tasks of cotton leaves between different cultivars. A similar phenomenon that which highly complex architectures had poor performance in regression tasks also occurred in the previous study [51].

### 3.7. Comparison between the Effect of Different Dataset Size on Fine-Tuning

The CNN1 with one convolution layer was chosen as the optimal architecture, and the effect of small dataset size on the performance of fine-tuned CNN1 models was compared. The model training process remained consistent with Section 2.4.3 except for the batch size change. Considering that when the small dataset size was just ten percent of the original calibration set, the number of samples was too small, which tends to cause over-fitting issues, so the batch size in the training process was adjusted to 32. The fine-tuned CNN1 models using dataset with different dataset sizes are shown in Table 8. No matter for the transfer task from LMY24 to XLZ53 or from XLZ53 to LMY24, with the dataset size used for fine-tuning increased, the performance of the fine-tuned model was gradually optimized, and the RMSE of the prediction set decreased. When LMY24 was the source domain, and the dataset size used in fine-tuning reached 50% of the original calibration set, the RMSE of the prediction set was just 8.1% higher than that based on the whole calibration set, achieving a satisfactory result. A similar phenomenon was observed in the transfer task from XLZ53 to LMY24. When half the samples in the calibration set were used in fine-tuning, the RMSE of the prediction set was only 4.1% higher than that using the whole samples in the calibration set, which suggested that fine-tuning with a relatively small dataset was capable of performing a satisfactory transfer.

## 4. Discussion

**Figure 5:**
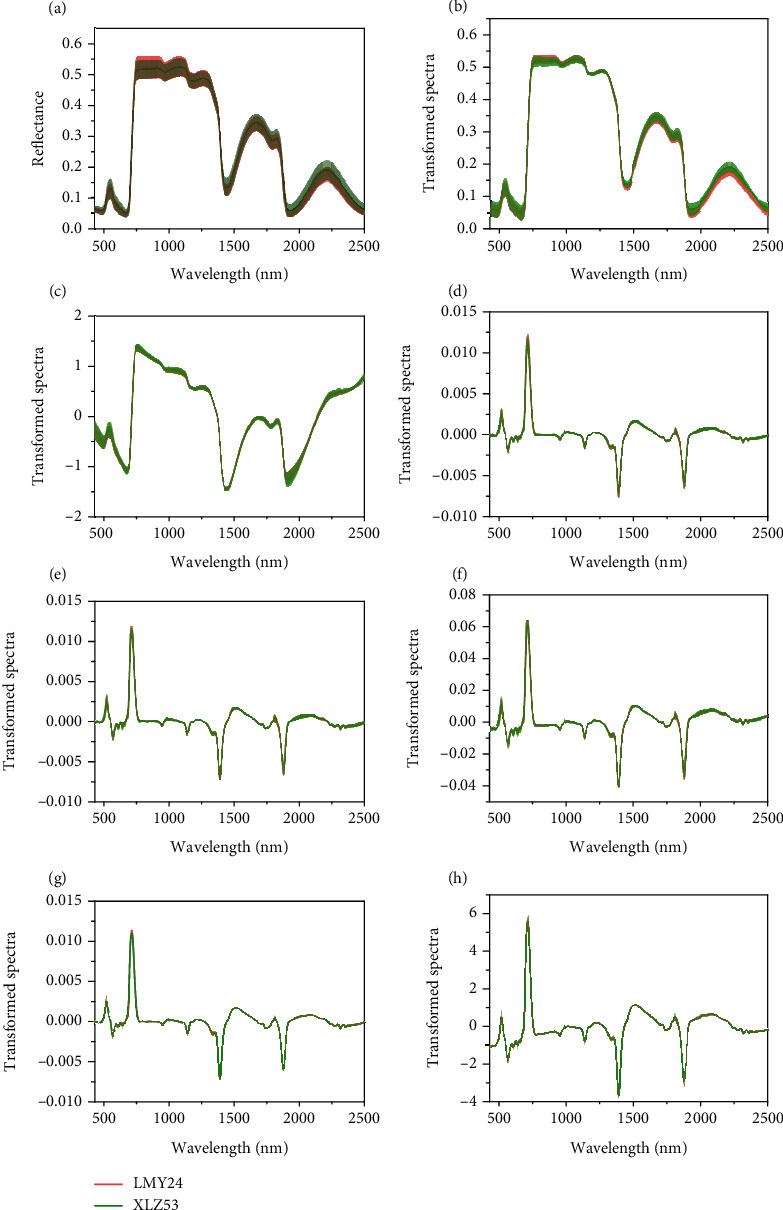
The average reflectance and the average transformed spectra with standard deviation (SD) for two cotton varieties. (a) raw spectra; (b) MSC; (c) SNV; (d) FD; (e) MSC+ FD; (f) SNV + FD; (g) FD + MSC; (h) FD + SNV.

Preprocessing can remove the background information and noises and keep useful sample-related information as far as possible, which is essential for establishing reliable and stable models. As shown in Figure 5, the average spectra without any preprocessing had a large deviation in reflectance, and a gap existed between the curves of LMY24 and XLZ53. The standard deviation of the transformed spectra was reduced after MSC pretreatment. SNV pretreatment also resulted in a similar reduction in standard deviation, and the gaps in spectra curves of the two cultivars narrowed. Besides, in the curves with FD preprocessing, the average curves and standard deviation of LMY24 and XLZ53 cultivars mostly overlapped. This phenomenon was also observed in other transformed spectra ever processed by FD. The spectral differences between varieties caused by non-cultivars-related factors were minimized to a maximum extent, indicating that FD pretreatment method has a strong ability to remove noise and retain information related to components in the cotton leaves of different varieties. However, it cannot be easy to intuitively and quantitatively analyze which combination was better just from the images of transformed spectra files. Therefore, we compared their influence on the model through modeling. As shown in Tables 3 and 4, FD + SNV was superior to others. Some studies discussed the advantages of spectral preprocessing methods for improving generalization ability [14, 52]. SNV showed excellent performance in cross-domain prediction and narrowed the gap between the spectral curves [14]. FD + SNV was shown effective in calibration transfer across different datasets [52]. The above results are consistent with the relatively better modeling results based on FD + SNV preprocessed spectra in this research.

Transfer learning can solve the problem that a model built on one dataset cannot be effectively applied to another dataset. Fine-tuning is one of the effective deep transfer learning methods. In this study, the optimal preprocessing was combined with transfer learning to detect the Chl content of leaves of two different cotton varieties. Results show that fine-tuning based on a simple neural network can effectively achieve a well-performed prediction across domains of various samples. In the study of [26], deep transfer learning was used for the calibration transfer of models between different instruments. Fine-tuned CNN based on a small dataset could achieve a satisfactory prediction for the slave instrument. Moreover, the studies investigated by Wu et al. [15] and Zhang et al. [53] both demonstrated the great capability of fine-tuned CNN to make the spectra knowledge of source domain transferrable to the target domain.

To further verify the proposed method's superiority, the presented approach's performance with conventional calibration and transfer methods was compared. The results of PLS models based on spectra that have been transformed by DS [20] and transfer component analysis (TCA) [54] were provided. The spectra have been preprocessed by FD + SNV, and the dataset division and PLS modeling were kept the same as those in Section 3.4. It is worth noting that in the process of DS, standard samples were randomly selected for three times (named DS1, DS2, and DS3) to investigate the influence of standard sample selection on the model. At each time, one hundred samples were selected from the source and target domain and were used for transformation matrix calculation. Then, all the spectra of the target domain were transformed based on the transformation matrix. After completing the corresponding spectra transformation, PLS models based on the spectra of the source domain were built and then used for target domain prediction. The results are shown in Table 9. It can be found that the prediction performance of the DS model varied when standard samples were selected differently. Moreover, the prediction performance for the three datasets of the target domain after TCA conversion was better than that after DS transformation. However, the results of these two methods are not as good as those based on the method combining preprocessing and fine-tuning.

Spectral preprocessing contributes to diminishing the difference in spectra, and deep transfer learning improves the ability to learn spectral features. The combination of these two approaches realizes effective calibration transfer between different domains. This study discussed the feasibility of the proposed method based on multiple batches of two varieties of cotton leaves. However, considering the high cost of acquiring the labeled data, research on improving the generalization performance of the model based on small datasets needs to be strengthened. Besides, the data distribution between the source and target domains needs to be considered. That means that how to choose samples with a reasonable data distribution for fine-tuning is the most time-saving and labor-saving still need to be further explored.

## 5. Conclusion

The development of spectral signal preprocessing equipped with deep transfer learning presents a new approach for model transfer between different domains. In this study, we investigated the potential of using spectral preprocessing and a pretrained CNN model to determine Chl content in cotton leaves. The success of the combination of FD and SNV in improving the transferable performance of PLS and SVR models between two cotton varieties provides an effective and standard-free approach for calibration transfer. The CNN was designed based on preprocessed spectra and further fine-tuned using spectra of another cotton cultivar. In the transfer task from the cultivar LMY24 to XLZ53, the transferred model obtained the RMSE of 2.505, 3.248, and 3.020 for the calibration, validation, and prediction set of the target domain. Similarly, in the transfer task from the cultivar XLZ53 to LMY24, the model achieved a good result, with the RMSE of 2.708, 3.332, and 3.460 for the three datasets of the target domain. The model combining spectral preprocessing and deep transfer learning obtained a good result, demonstrating the effectiveness of the proposed approach. In future studies, more cotton cultivars and more variations in spectra will be considered to improve the robustness of the models further.

## Figures and Tables

**Table 1:** Statistical information of cotton leaves in the calibration and prediction sets.

Sample set	Number	Range	Mean	Standard deviation
Cal	1026	9.690-56.069	32.893	8.300
Pre	512	10.750-54.962	32.848	8.244

**Table 2:** Prediction results for both varieties of cotton.

Model	R^2^_CV_	RMSECV	R^2^_P_	RMSEP
PLS	0.806	3.651	0.768	3.996
SVR	0.879	2.88	0.822	3.472

**Table 3:** Results of models built on different preprocessed spectra when LMY 24 was the source domain.

Model	Pretreatment	LMY 24		XLZ53	
		R^2^_CV_	RMSECV	R^2^_P_	RMSEP
PLS	None	0.801	3.646	0.734	4.459
	MSC	0.789	3.759	0.726	4.593
	SNV	0.793	3.713	0.681	5.428
	FD	0.824	3.424	0.671	5.091
	MSC+ FD	0.810	3.561	0.772	4.021
	SNV+ FD	0.789	3.752	0.773	4.140
	FD + MSC	0.797	3.680	0.794	3.839
	FD + SNV	**0.827**	**3.399**	**0.838**	**3.289**
SVR	None	0.867	2.981	0.700	4.572
	MSC	0.878	2.849	0.602	5.260
	SNV	0.864	3.008	0.626	5.104
	FD	0.874	2.897	0.681	4.713
	MSC+ FD	0.872	2.915	0.511	5.836
	SNV+ FD	0.882	2.808	0.705	4.530
	FD + MSC	**0.871**	**2.935**	**0.732**	**4.317**
	FD + SNV	**0.874**	**2.902**	**0.724**	**4.384**

The numbers are bolded to highlight models with relatively good results.

**Table 4:** Results of models built on different preprocessed spectra when XLZ53 was the source domain.

Model	Pretreatment	XLZ53		LMY 24	
		R^2^_CV_	RMSECV	R^2^_P_	RMSEP
PLS	None	0.834	3.408	0.578	5.552
	MSC	0.809	3.656	0.577	5.562
	SNV	0.793	3.799	0.548	5.846
	FD	0.829	3.455	0.573	5.659
	MSC+ FD	0.837	3.372	0.651	5.725
	SNV+ FD	0.835	3.392	0.639	5.135
	FD + MSC	0.837	3.376	0.637	5.209
	FD + SNV	**0.844**	**3.308**	**0.681**	**4.731**
SVR	None	0.896	2.687	0.618	5.047
	MSC	0.902	2.611	0.635	4.934
	SNV	0.893	2.725	0.597	5.182
	FD	0.898	2.667	0.639	4.907
	MSC+ FD	0.896	2.686	0.638	4.913
	SNV+ FD	0.904	2.589	0.567	5.375
	FD + MSC	0.897	2.679	0.636	4.928
	FD + SNV	**0.889**	**2.777**	**0.647**	**4.853**

The numbers are bolded to highlight models with relatively good results.

**Table 5:** Regression results using fine-tuned CNN and conventional models.

Source/target domain	Pretreatment	Model	Calibration set^a^		Validation set^b^		Prediction set^c^	
			R^2^	RMSE	R^2^	RMSE	R^2^	RMSE
LMY24/XLZ53	None	PLS	0.719	4.792	0.663	5.596	0.696	5.235
		SVR	0.733	4.297	0.629	5.113	0.667	4.834
		Fine-tuned CNN	0.850	3.225	0.796	3.786	0.842	3.327
		Fine-tuned CNN using a smaller set	0.885	2.901	0.802	3.730	0.811	3.643
	FD + SNV	PLS	0.821	3.600	0.754	4.359	0.777	4.098
		SVR	0.761	4.065	0.662	4.877	0.761	4.090
		Fine-tuned CNN	**0.909**	**2.505**	**0.850**	**3.248**	**0.870**	**3.020**
		Fine-tuned CNN using a smaller set	**0.914**	**2.451**	**0.815**	**3.613**	**0.853**	**3.207**
XLZ53/LMY24	None	PLS	0.538	5.749	0.586	5.423	0.559	5.810
		SVR	0.537	5.537	0.561	5.429	0.506	5.759
		Fine-tuned CNN	0.850	3.156	0.746	4.129	0.757	4.036
		Fine-tuned CNN using a smaller set	0.734	4.235	0.672	4.691	0.689	4.568
	FD + SNV	PLS	0.637	5.140	0.630	5.086	0.636	5.300
		SVR	0.654	4.909	0.664	4.928	0.578	5.445
		Fine-tuned CNN	**0.889**	**2.708**	**0.835**	**3.332**	**0.822**	**3.460**
		Fine-tuned CNN using a smaller set	**0.892**	**2.697**	**0.822**	**3.458**	**0.796**	**3.703**

^a^Calibration set means the calibration set of the target domain; ^b^Validation set means the validation set of the target domain; ^c^prediction set means the prediction set of the target domain; the numbers are bolded to highlight models with relatively good results.

**Table 6:** The CNN architectures used in the experiment.

Layer		CNN1	CNN2	CNN3	CNN4	Alexnet	VGGNet-9
Input		1 × 2071	1 × 2071	1 × 2071	1 × 2071	1 × 2071	1 × 2071
Convolution	1	32 kernels in size 1 × 3 with max pooling	32 kernels in size 1 × 3 with max pooling	32 kernels in size 1 × 3 with max pooling	32 kernels in size 1 × 3 with max pooling	96 kernels in size 1 × 11 with max pooling	64 kernels in size 1 × 5
	2	—	32 kernels in size 1 × 3 with max pooling	32 kernels in size 1 × 3 with max pooling	32 kernels in size 1 × 3 with max pooling	256 kernels in size 1 × 5 with max pooling	64 kernels in size 1 × 3 with max pooling
	3	—	—	32 kernels in size 1 × 3 with max pooling	32 kernels in size 1 × 3 with max pooling	384 kernels in size 1 × 3 with max pooling	128 kernels in size 1× 3
	4	—	—	—	32 kernels in size 1 × 3 with max pooling	384 kernels in size 1 × 3	128 kernels in size 1 × 3 with max pooling
	5	—	—	—	—	256 kernels in size 1 × 3	256 kernels in size 1 × 3
	6	—	—	—	—	—	256 kernels in size 1 × 3
	7	—	—	—	—	—	256 kernels in size 1 × 3 with max pooling
Fully connected	1	512 nodes, ReLu	512 nodes, ReLu	512 nodes, ReLu	512 nodes, ReLu	4096 nodes, ReLu	4096 nodes, ReLu
	2	32 nodes, ReLu	32 nodes, ReLu	32 nodes, ReLu	32 nodes, ReLu	4096 nodes, ReLu	4096 nodes, ReLu
Output		1 node	1 node	1 node	1 node	1 node	1 node

**Table 7:** Results of fine-tuned models using different CNN architectures.

Source/target domain	Model	Calibration set^a^		Validation set^b^		Prediction set^c^	
		R^2^	RMSE	R^2^	RMSE	R^2^	RMSE
LMY24/XLZ53	CNN1	0.921	2.336	0.853	3.217	0.880	2.903
	CNN2	0.909	2.505	0.850	3.248	0.870	3.020
	CNN3	0.910	2.501	0.821	3.550	0.855	3.184
	CNN4	0.892	2.739	0.840	3.356	0.855	3.186
	AlexNet	0.877	2.923	0.848	3.269	0.852	3.222
	VGGNet-9	0.897	2.672	0.819	3.570	0.840	3.348
XLZ53/LMY24	CNN1	0.891	2.691	0.828	3.399	0.820	3.476
	CNN2	0.907	2.454	0.828	3.397	0.818	3.494
	CNN3	0.910	2.444	0.836	3.319	0.818	3.497
	CNN4	0.898	2.599	0.826	3.414	0.817	3.508
	AlexNet	0.864	3.003	0.813	3.549	0.819	3.489
	VGGNet-9	0.891	2.693	0.813	3.550	0.816	3.509

^a^Calibration set means the calibration set of the target domain; ^b^validtion set means the validation set of the target domain; ^c^prediction set means the prediction set of the target domain.

**Table 8:** Results of fine-tuned CNN1 models using a dataset with different size.

Source/target domain	Dataset size^s^	Calibration set^a^		Validation set^b^		Prediction set^c^	
		R^2^	RMSE	R^2^	RMSE	R^2^	RMSE
LMY24/XLZ53	10%	0.829	3.718	0.706	4.550	0.656	4.807
	20%	0.868	3.012	0.789	3.851	0.810	3.651
	30%	0.873	2.804	0.802	3.729	0.832	3.433
	40%	0.859	3.028	0.817	3.586	0.838	3.372
	50%	0.852	3.237	0.832	3.441	0.842	3.330
	60%	0.866	3.058	0.831	3.454	0.841	3.333
	70%	0.871	2.961	0.838	3.380	0.844	3.305
	80%	0.856	3.153	0.837	3.383	0.846	3.286
	90%	0.865	3.086	0.851	3.244	0.852	3.224
	100%	0.879	2.895	0.841	3.346	0.864	3.083
XLZ53/LMY24	10%	0.864	3.484	0.712	4.397	0.701	4.481
	20%	0.872	3.238	0.753	4.076	0.729	4.263
	30%	0.830	3.579	0.773	3.901	0.753	4.073
	40%	0.832	3.405	0.817	3.505	0.792	3.737
	50%	0.837	3.387	0.817	3.507	0.801	3.652
	60%	0.868	3.004	0.795	3.707	0.805	3.621
	70%	0.870	3.023	0.821	3.466	0.804	3.626
	80%	0.836	3.335	0.821	3.471	0.814	3.533
	90%	0.840	3.283	0.813	3.546	0.815	3.528
	100%	0.852	3.131	0.822	3.459	0.817	3.507

^s^Dataset size means the percentage of small dataset size participating in fine-tuning to the dataset size of the original calibration set. ^a^Calibration set means the calibration set of the target domain; ^b^validation set means the validation set of the target domain; ^c^prediction set means the prediction set of the target domain.

**Table 9:** The prediction results of PLS models using DS and TCA transformation.

Source/target domain	Method	Calibration set^a^		Validation set^b^		Prediction set^c^	
		R^2^	RMSE	R^2^	RMSE	R^2^	RMSE
LMY24/XLZ53	DS 1	0.598	5.485	0.651	5.263	0.583	5.589
	DS 2	0.546	5.902	0.507	6.198	0.565	5.812
	DS 3	0.623	5.789	0.687	5.271	0.623	5.935
	TCA	0.811	3.652	0.741	4.427	0.788	3.936
XLZ53/LMY24	DS 1	0.466	6.821	0.462	6.950	0.499	6.648
	DS 2	0.449	6.948	0.461	6.887	0.425	6.989
	DS 3	0.388	7.456	0.289	8.169	0.452	6.885
	TCA	0.600	5.446	0.591	5.406	0.600	5.654

^a^Calibration set means the calibration set of the target domain; ^b^validation set means the validation set of the target domain; ^c^prediction set means the prediction set of the target domain.

## Data Availability

The data used to support the findings of this study are available from the corresponding author upon request.
